# Development and validation of a rapid *Listeria monocytogenes* loop-mediated isothermal amplification assay in soft cheese and raw pet food

**DOI:** 10.3389/fmicb.2026.1920965

**Published:** 2026-09-01

**Authors:** Beilei Ge, Kelly J. Domesle, Leticia Mallmann, Madelyn Springer, Xiaohong Deng, Hee Jin Kwon, Eduardo Ximenes, Yi Chen

**Affiliations:** 1 Office of Applied Science, Center for Veterinary Medicine, U.S. Food and Drug Administration, Laurel, MD, United States; 2 Department of Environmental and Occupational Health, School of Public Health, Indiana University, Bloomington, IN, United States; 3 Joint Institute for Food Safety and Applied Nutrition, University of Maryland, College Park, MD, United States; 4 U.S. Food and Drug Administration Human Foods Program, College Park, MD, United States

**Keywords:** animal food, *hlyA*, human food, *Listeria monocytogenes*, loop-mediated isothermal amplification, raw pet food, soft cheese

## Abstract

**Introduction:**

Rapid screening of *Listeria monocytogenes* in human and animal foods is imperative for improving laboratory efficiency and safeguarding food supply.

**Methods:**

We developed a loop-mediated isothermal amplification (LAMP) assay targeting the *L. monocytogenes hlyA* gene (listeriolysin O) and performed single-laboratory validation in representative human (soft cheese) and animal (raw pet food) food matrices. This study followed the U.S. Food and Drug Administration’s (FDA) Microbiological Method Validation Guidelines and used the FDA’s *Bacteriological Analytical Manual* (BAM) method as a reference.

**Results:**

The assay produced accurate results (positive and negative) by analyzing the inclusivity and exclusivity panels comprising 50 *L. monocytogenes*, 20 non-*L. monocytogenes Listeria*, and 31 non-*Listeria*, with a detection limit of 100 cells per reaction, when using *L. monocytogenes* American Type Culture Collection (ATCC) 19115. Both soft cheese and raw pet food validations yielded satisfactory results, with the relative levels of detection (RLODs) falling within the acceptability limit (1.5) for a paired study. This suggests that LAMP analysis of 48 h enrichments was as sensitive as the BAM culture method in detecting low-level *L. monocytogenes* contamination in both matrices, with reliable screening results available at least 3 days sooner.

**Discussion:**

This study demonstrates the potential of this rapid and reliable LAMP assay for routine screening of *L. monocytogenes* in soft cheese and raw pet food and, upon further validation, additional human and animal foods to ensure product safety and protect public health.

## 1 Introduction

The Gram-positive, facultative anaerobic bacterium *Listeria monocytogenes* is a significant foodborne pathogen around the globe, due in part to its high case-fatality rate (20–30%) in pregnant women, the elderly, and other immunocompromised individuals (WHO, 2018). In the United States, *L. monocytogenes* causes an estimated 1,250 illnesses and 172 deaths annually (Scallan Walter et al., 2025). The infection rate has remained relatively stable at approximately 0.3 incidence per 100,000 population, with a hospitalization rate of 93%, underscoring the severity of listeriosis (CDC, 2026a). Multistate listeriosis outbreaks linked to diverse food vehicles, such as soft cheeses, deli meats, prepared meals, smoked fish, and fresh produce, have occurred (CDC, 2026b). With the recent trend of pet owners feeding their pets raw meat-based diets (APPA, 2026), an increase in recalls due to *L. monocytogenes* in raw pet food has been documented (FDA, 2026a), highlighting a broad safety risk in human and animal foods.

Multifaceted approaches are imperative to address the public health risks associated with *L. monocytogenes* infections (FDA, 2008; USDA, 2014). The U.S. Food and Drug Administration (FDA) and the U.S. Department of Agriculture (USDA) built regulatory frameworks, including the Food Safety Modernization Act (FSMA) Preventive Controls rules promulgated by the FDA for human and animal foods, which require food facilities to implement food safety plans that include environmental monitoring and product testing for *L. monocytogenes* and other pathogens (FDA, 2015a,b).

Culture-based methods remain the gold standard for microbiological analysis of *L. monocytogenes* in foods, for instance, the FDA’s *Bacteriological Analytical Manual* (BAM) Chapter 10 (Hitchins et al., 2022), the USDA’s *Microbiology Laboratory Guidebook* (MLG) Method 8 (USDA, 2024), and International Organization for Standardization (ISO) 11290-1/2 (ISO, 2017a,b), which detail procedures for *L. monocytogenes* detection and enumeration. These methods involve selective enrichment in buffered *Listeria* enrichment broth (BLEB), bioMérieux’s *Listeria* Phage Technology (LPT) broth (bioMérieux, Durham, NC, United States), or half-Fraser broth and Fraser broth, followed by selective plating on esculin-based *Listeria* agars (e.g., Oxford and PALCAM) and/or chromogenic agars (e.g., agar *Listeria* according to Ottaviani and Agosti [ALOA] and RAPID’*L.mono* [Bio-Rad, Hercules, CA, United States]), and biochemical, serological, or mass spectrometry-based confirmation, with the entire process taking 2 weeks or longer. Molecular screening methods have been developed to provide early warning, with several commercial kits obtaining the AOAC INTERNATIONAL (AOAC) Official Methods of Analysis (OMA) status, including real-time polymerase chain reaction (PCR) and loop-mediated isothermal amplification (LAMP) (Andrews and Hammack, 2023). Nonetheless, open-source molecular assays that have been vigorously validated in various human and animal foods and are compatible with the FDA BAM workflow are scarce.

As a promising alternative to real-time PCR, LAMP is now at the forefront of isothermal nucleic acid amplification technology (iNAAT), with the two main advantages of eliminating the need for thermal cyclers and possessing high tolerance to matrix inhibitors (Notomi et al., 2000; ISO, 2026; Yang et al., 2018). LAMP employs 4–6 primers that recognize 6–8 distinct regions of the target nucleic acid sequence, ensuring high specificity (Notomi et al., 2000). Amplification occurs through back a strand-displacing DNA polymerase (e.g., *Bst*), producing large amounts of DNA with characteristic stem-loop structures rather efficiently (Notomi et al., 2000). LAMP amplicons can be detected through multiple chemistries, including fluorescence, bioluminescence, colorimetry, turbidity, electrochemistry, or lateral flow (ISO, 2026).

This study aimed to develop a rapid, specific, and sensitive *L. monocytogenes* LAMP assay and validate it in representative human (soft cheese) and animal (raw pet food) foods using the FDA’s BAM culture method as a reference. The single-laboratory validation (SLV) followed the FDA’s Microbiological Method Validation Guidelines (FDA, 2019), which align well with those of the AOAC and the ISO (AOAC, 2023; ISO, 2016).

## 2 Materials and methods

### 2.1 Bacterial strains and culture conditions

All bacterial strains used in this study were stored cryogenically at −80 °C in brain heart infusion (BHI) broth (BD, Sparks, MD, United States) with 20% glycerol and cultured on blood agar plates (BAP) (Thermo Fisher Scientific, Waltham, MA, United States) at 37 °C overnight. Specificity testing included an inclusivity panel (*n* = 50) and an exclusivity panel (*n* = 51) (Table 1). The 50 *L. monocytogenes* strains in the inclusivity panel belonged to various lineages (I, II, III, and N/A) and serotypes (1/2a, 1/2b, 1/2c, 3a, 3b, 3c, 4a, 4b, 4c, and N/A). The exclusivity panel comprised 20 non-*L. monocytogenes Listeria* strains representing *L. grayi*, *L. innocua*, *L. ivanovii*, *L. seeligeri*, and *L. welshimeri* and 31 non-*Listeria* strains including the genera *Bacillus*, *Bifidobacterium*, *Citrobacter*, *Enterococcus*, *Escherichia*, *Klebsiella*, *Lacticaseibacillu*s, *Salmonella*, *Serratia*, *Shigella*, *Staphylococcus*, *Streptococcus*, and *Yersinia*.

**Table 1:** Bacterial strains (*n* = 101) used in inclusivity and exclusivity panels for *Listeria monocytogenes* loop-mediated isothermal amplification (LAMP) specificity testing and results.

Strain ID^a^	Serotype for *L. monocytogenes* or genus for others	Lineage for *L. monocytogenes* or species for others	Origin	Source^b^	LAMP *T_p_* (min)	LAMP *T_a_* (°C)
*Listeria monocytogenes* inclusivity panel (*n* = 50)						
C1-056	1/2a	II	Clinical	FDA/HFP	6.7	82.1
J1-101	1/2a	II	Clinical	FDA/HFP	8.4	82.3
J1-105	1/2a	II	Clinical	FDA/HFP	9.8	82.3
J2-003	1/2a	II	Animal	FDA/HFP	11.7	82.2
J2-031	1/2a	II	Animal	FDA/HFP	8.3	82.3
J2-054	1/2a	II	Animal	FDA/HFP	7.7	82.3
J2-063	1/2a	II	Animal	FDA/HFP	8.4	82.3
J2-066	1/2a	II	Animal	FDA/HFP	7.4	82.1
N3-031	1/2a	II	Food	FDA/HFP	7.7	82.2
R2-499	1/2a	II	Clinical	FDA/HFP	7.7	82.2
R2-603	1/2a	II	Food	FDA/HFP	9.4	82.2
J1-177	1/2b	I	Clinical	FDA/HFP	10.7	82.7
J2-035^a^	1/2b	I	Animal	FDA/HFP	10.6	83.0
J2-064	1/2b	I	Animal	FDA/HFP	11.7	82.8
R2-502	1/2b	I	Food	FDA/HFP	13.3	82.8
R2-503	1/2b	I	Clinical	FDA/HFP	9.6	82.8
J1-094^a^	1/2c	II	Clinical	FDA/HFP	8.5	82.8
C1-115	3a	II	Clinical	FDA/HFP	6.48	82.1
M1-004	3a	II	Clinical	FDA/HFP	11.2	82.1
J1-169	3b	I	Clinical	FDA/HFP	14.0	82.6
J1-049^a^	3c	I	Clinical	FDA/HFP	13.2	83.1
J1-031	4a	III	Clinical	FDA/HFP	9.1	82.0
J1-168	4a	III	Clinical	FDA/HFP	12.2	82.3
W1-112^a^	4a	III	Unknown	FDA/HFP	22.5	82.6
C1-122^a^	4b	I	Clinical	FDA/HFP	15.0	83.3
H7355	4b	I	Clinical	FDA/HFP	8.6	82.6
H7961	4b	I	Food	FDA/HFP	8.6	82.5
J1-002	4b	I	Food	FDA/HFP	10.0	82.5
J1-110	4b	I	Food	FDA/HFP	9.5	82.7
J1-116	4b	I	Clinical	FDA/HFP	11.2	82.8
J1-119	4b	I	Clinical	FDA/HFP	11.8	82.8
J1-123	4b	I	Clinical	FDA/HFP	11.0	82.7
J1-126	4b	I	Clinical	FDA/HFP	13.2	82.6
J1-158^a^	4b	III	Animal	FDA/HFP	17.4	83.0
J1-220	4b	I	Clinical	FDA/HFP	13.2	82.7
J1-225	4b	I	Clinical	FDA/HFP	12.0	82.7
J1776	4b	I	Clinical	FDA/HFP	9.5	82.6
J1816	4b	I	Environmental	FDA/HFP	8.7	82.7
N3-008	4b	I	Food	FDA/HFP	10.2	82.8
N3-010	4b	I	Food	FDA/HFP	10.6	82.7
N3-013	4b	I	Clinical	FDA/HFP	10.9	82.5
N3-022	4b	I	Clinical	FDA/HFP	17.8	82.6
R2-500	4b	I	Food	FDA/HFP	13.7	82.4
R2-501	4b	I	Clinical	FDA/HFP	11.6	82.5
R2-578	4b	I	Clinical	FDA/HFP	11.5	82.8
R2-583	4b	I	Clinical	FDA/HFP	20.8	82.7
W1-111^a^	4c	III	Unknown	FDA/HFP	25.4	84.9
W1-110	4c	III	Unknown	FDA/HFP	11.2	82.3
C1-113	N/A	N/A	Food	FDA/HFP	6.1	82.0
R2-219	N/A	N/A	Environmental	FDA/HFP	9.7	82.7
Non-*L. monocytogenes Listeria* exclusivity panel (*n* = 20)						
Lgy	*Listeria*	*grayi*	Unknown	FDA/HFP	Blank	Blank
40022	*Listeria*	*innocua*	Unknown	FDA/HFP	Blank	Blank
Li	*Listeria*	*innocua*	Cheese	FDA/HFP	Blank	Blank
Ls-007	*Listeria*	*innocua*	Milk	FDA/HFP	Blank	Blank
Ls-010	*Listeria*	*innocua*	Cheese	FDA/HFP	Blank	Blank
Ls-028	*Listeria*	*innocua*	Cheese	FDA/HFP	Blank	Blank
Ls-054	*Listeria*	*innocua*	Milk	FDA/HFP	Blank	Blank
Ls-127	*Listeria*	*innocua*	Shrimp	FDA/HFP	Blank	Blank
40016	*Listeria*	*ivanovii*	Unknown	FDA/HFP	Blank	Blank
DSM 20750	*Listeria*	*ivanovii*	Sheep	FDA/HFP	Blank	Blank
Liv	*Listeria*	*ivanovii*	Clinical	FDA/HFP	Blank	Blank
LM4-3B	*Listeria*	*ivanovii*	Animal	FDA/HFP	Blank	Blank
Ls-123	*Listeria*	*ivanovii*	Unknown	FDA/HFP	Blank	Blank
35967	*Listeria*	*seeligeri*	Soil	FDA/HFP	Blank	Blank
40006	*Listeria*	*seeligeri*	Unknown	FDA/HFP	Blank	Blank
40019	*Listeria*	*seeligeri*	Unknown	FDA/HFP	Blank	Blank
40023	*Listeria*	*seeligeri*	Unknown	FDA/HFP	Blank	Blank
BA-84	*Listeria*	*welshimeri*	Cheese	FDA/HFP	Blank	Blank
Ls-156	*Listeria*	*welshimeri*	Sediment	FDA/HFP	Blank	Blank
Lw	*Listeria*	*welshimeri*	Milk	FDA/HFP	Blank	Blank
Non-*Listeria* exclusivity panel (*n* = 31)						
ATCC 23350	*Bacillus*	*amyloliquefaciens*	Soil	ATCC	Blank	Blank
ATCC 23842	*Bacillus*	*amyloliquefaciens*	Unknown	ATCC	Blank	Blank
BAC0013	*Bacillus*	*cereus*	Lab control	FDA/HFP	Blank	Blank
BAC00216	*Bacillus*	*licheniformis*	Lab control	FDA/HFP	Blank	Blank
BAC00213	*Bacillus*	*megaterium*	Cosmetics	FDA/HFP	Blank	Blank
BAC00270	*Bacillus*	*pumilus*	Lab control	FDA/HFP	Blank	Blank
NES 6	*Bacillus*	*subtilis*	Soil	FDA/HFP	Blank	Blank
ATCC 10792	*Bacillus*	*thuringiensis*	Animal	ATCC	Blank	Blank
BB-12	*Bifidobacterium*	*animalis* subsp*. lactis*	Lab control	FDA/HFP	Blank	Blank
NES 12	*Citrobacter*	*koseri*	Clinical	FDA/HFP	Blank	Blank
40003	*Enterococcus*	*faecalis*	Food	FDA/HFP	Blank	Blank
ATCC 19434	*Enterococcus*	*faecium*	Lab control	ATCC	Blank	Blank
NES 11	*Escherichia*	*coli*	Clinical	FDA/HFP	Blank	Blank
NES 20	*Escherichia*	*hermannii*	Clinical	FDA/HFP	Blank	Blank
NES 2	*Klebsiella*	*aerogenes*	Clinical	FDA/HFP	Blank	Blank
ATCC 393	*Lacticaseibacillus*	*casei*	Cheese	ATCC	Blank	Blank
LGG	*Lacticaseibacillus*	*rhamnosus*	Clinical	FDA/HFP	Blank	Blank
NES 51	*Salmonella*	*enteritidis*	Food	FDA/HFP	Blank	Blank
NES 52	*Salmonella*	*typhimurium*	Food	FDA/HFP	Blank	Blank
NES 18	*Serratia*	*ficaria*	Food	FDA/HFP	Blank	Blank
SAFE 109	*Shigella*	*flexneri*	Clinical	FDA/HFP	Blank	Blank
SAFE 108	*Shigella*	*sonnei*	Clinical	FDA/HFP	Blank	Blank
ATCC 25923	*Staphylococcus*	*aureus*	Clinical	FDA/HFP	Blank	Blank
40001	*Staphylococcus*	*epidermidis*	Clinical	FDA/HFP	Blank	Blank
ATCC 29663	*Staphylococcus*	*intermedius*	Animal	FDA/HFP	Blank	Blank
ATCC 15305	*Staphylococcus*	*saprophyticus*	Clinical	ATCC	Blank	Blank
136	*Staphylococcus*	*sciuri*	Food	FDA/HFP	Blank	Blank
ATCC 13813	*Streptococcus*	*agalactiae*	Lab control	ATCC	Blank	Blank
40002	*Streptococcus*	*pyogenes*	Clinical	FDA/HFP	Blank	Blank
Ye37	*Yersinia*	*enterocolitica*	Unknown	FDA/HFP	Blank	Blank
Yp1313	*Yersinia*	*pseudotuberculosis*	Unknown	FDA/HFP	Blank	Blank

a For seven strains, blank *T_p_* and/or blank *T_a_* were obtained by LAMP when testing the initial inclusivity templates at approximately 10^0^–10^2^ cells/reaction. The data presented were obtained from further testing using serial dilutions of approximately 10^2^-10^3^ cells/reaction.

b ATCC, American Type Culture Collection; FDA/HFP, U.S. Food and Drug Administration, Human Foods Program.

Additionally, *L. monocytogenes* ATCC 19115 (American Type Culture Collection, Manassas, VA, United States), a serotype 4b clinical strain, was used for sensitivity evaluation. *L. monocytogenes* CFSAN011016, isolated from fresh cheese curd and belonging to serotype 1/2b, was used for validation in soft cheese, and *L. monocytogenes* ATCC 19111, a serotype 1/2a strain originally isolated from poultry, was used for validation in raw pet food. The *L. monocytogenes* CFSAN011016 strain was obtained from the FDA’s Human Foods Program (HFP)’s stock culture collection.

### 2.2 LAMP assay design and optimization

**Figure 1:**
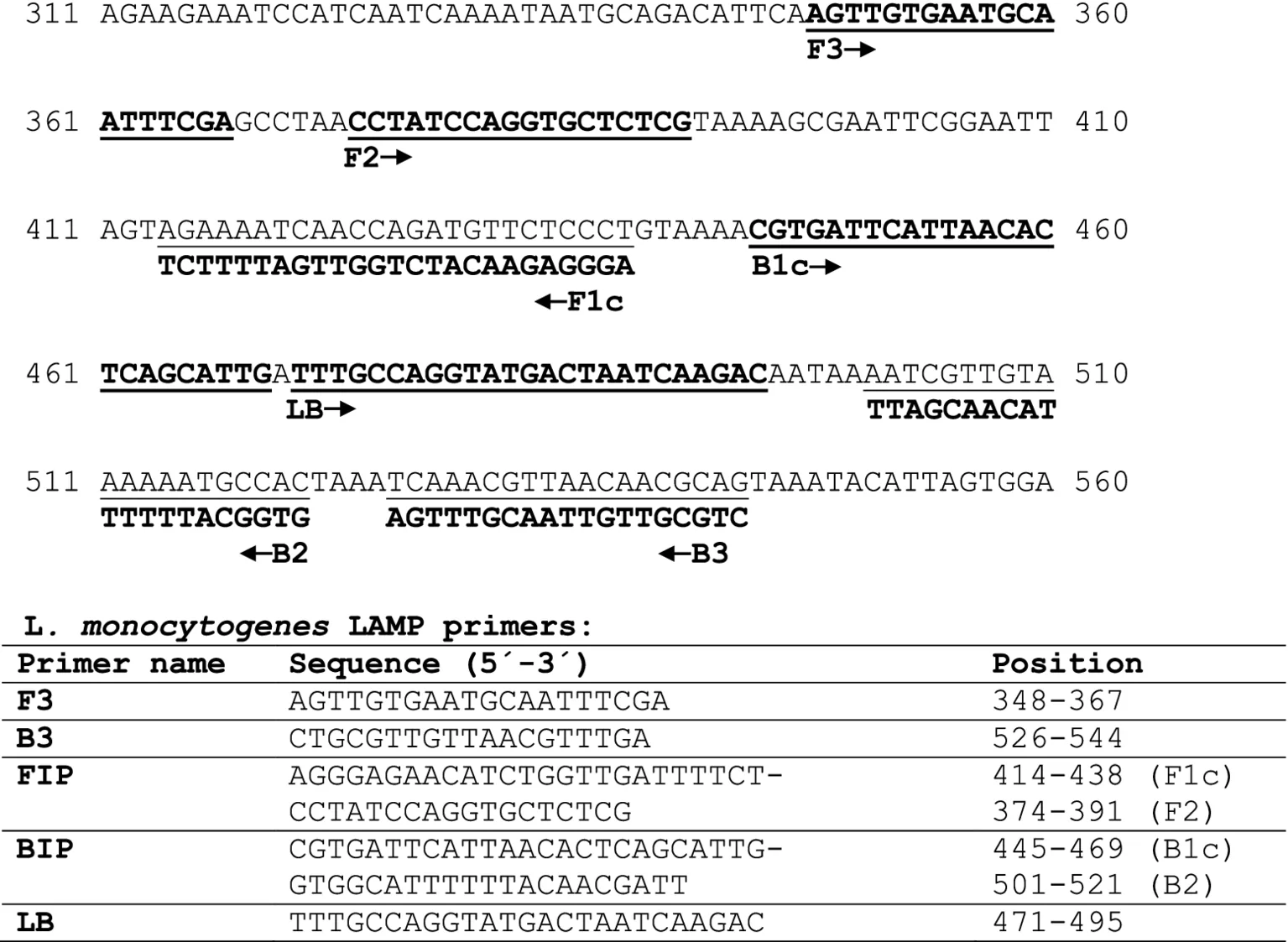
Sequence alignments illustrating positions of five *Listeria monocytogenes* loop-mediated isothermal amplification (LAMP) primers (F3, B3, FIP [F1c + F2], BIP [B1c + B2], and LB) in the target *hlyA* gene. A partial sequence of the *L. monocytogenes* listeriolysin O gene, *hlyA* (GenBank accession number AL591824), is shown. F3 and B3 are the forward outer primer and backward outer primer, respectively; FIP and BIP are the forward inner primer and backward inner primer, respectively; LB is the backward loop primer.

The *L. monocytogenes* listeriolysin O-encoding *hlyA* gene (1,590 bp, extracted from the complete genome of *L. monocytogenes* EGD-e chromosome, serotype 1/2a, GenBank accession number AL591824 (Glaser et al., 2001)) was selected as the target for the LAMP primer design. A set of five primers (Figure 1), two outer (F3 and B3), two inner (FIP and BIP), and one loop (backward loop primer [LB]) that recognized seven target regions was designed using PrimerExplorer V5 (Eiken Chemical Co., Tokyo, Japan; https://primerexplorer.jp/lampv5e/). FIP consisted of a complementary sequence of F1 and a sense sequence of F2, without any linker sequence in between. Similarly, BIP was a direct combination of a complementary sequence of B1 and a sense sequence of B2. All LAMP primers were selected based on previously described criteria (Notomi et al., 2000; Nagamine et al., 2002) and the software developer’s recommendations (Eiken Chemical Co. Ltd., 2019).

The prototypic LAMP reaction mix (25 μL total) consisted of 1 × GspSSD2.0 Isothermal Mastermix (ISO-004; OptiGene Ltd., West Sussex, United Kingdom), 1 × primer mix (0.1 μM F3/B3, 1.8 μM FIP/BIP, and 1 μM LB; Integrated DNA Technologies, Coralville, Iowa, United States), and 5 μL of DNA template. A positive control (*L. monocytogenes* ATCC 19115 at *ca.* 10^4^ cells/reaction) and a no-template control (molecular grade water) were included. The LAMP reaction was carried out in a portable real-time fluorometer (Genie II, OptiGene Ltd.) using a run profile of amplification at 65 °C for 30 min and annealing from 98 °C to 80 °C at a ramp rate of 0.05 °C per second. Real-time fluorescence readings were acquired using the FAM channel, and time-to-positive values (peak ratios or *T_p_*; min) were obtained when fluorescence ratios reached the maximum values of the amplification rate curves. Annealing temperatures of LAMP amplicons (anneal peaks or *T_a_*; °C) were determined at the maximum values of anneal derivative curves. Samples were considered LAMP positive with *T_p_* ≤ 30 min and *T_a_* around 82.5 °C, reflecting specific amplification of the target *hlyA* gene.

Both the LAMP primer mix/ratio and reaction temperature were optimized. Primer mixes tested included OptiGene Standard (0.2 μM F3/B3, 0.8 μM FIP/BIP, and 0.4 μM LB), OptiGene High (0.2 μM F3/B3, 2 μM FIP/BIP, and 1 μM LB), NEB WarmStart (0.2 μM F3/B3, 1.6 μM FIP/BIP, and 0.4 μM LB; WarmStart LAMP Kit; New England Biolabs, Ipswich, MA, United States), and the prototypic one above (0.1 μM F3/B3, 1.8 μM FIP/BIP, and 1 μM LB). The reaction temperatures tested were 60 °C, 61 °C, 62 °C, 63 °C, 64 °C, and 65 °C, and a temperature gradient from 60 °C to 67 °C was used according to the Genie II instrument setup.

### 2.3 LAMP specificity and sensitivity

DNA templates for LAMP specificity (inclusivity and exclusivity) testing (Table 1) were prepared per the FDA guidelines (FDA, 2019). Briefly, several single colonies of the 50 *L. monocytogenes* inclusivity strains cultured on BAP were transferred to 5 mL of BHI broth and incubated at 37 °C overnight with shaking to reach growth limits. The cultures were serially diluted in 0.1% peptone water to obtain *ca.* 10^3^–10^5^ CFU/mL (i.e., 10^0^–10^2^ cells/reaction). Aliquots (450 μL) were used for automated DNA extraction using the BioSprint 96 One-For-All Vet Kit (INDICAl Inc., Orlando, FL, United States) on the KingFisher Flex-96 Purification System (Thermo Fisher Scientific). The final elution plate containing DNA templates (~100 μL) was sealed and stored at −20 °C. For the 51 non-*L. monocytogenes* exclusivity strains, DNA templates were prepared similarly, but the target level was the growth limit of the bacteria, which was approximately 10^9^ CFU/mL (i.e., 10^6^ cells/reaction). Concentrations of select specific templates were quantified using the Quant-iT Broad-Range dsDNA Assay Kit on a Qubit fluorometer (Thermo Fisher Scientific). Aliquots (5 μL) of each template were subjected to the LAMP assay with one replicate per strain.

LAMP sensitivity (limit of detection, LOD) was determined using 10-fold serial dilutions of *L. monocytogenes* ATCC 19115. DNA templates were prepared as described above, but with a series of concentrations ranging from 10^9^ to 10^3^ CFU/mL (i.e., 10^6^–10^0^ cells/reaction). Spread plate counting using the pour-plate method on plate count agar was performed to obtain the actual cell numbers. Aliquots (5 μL) of each template were tested by LAMP and repeated a total of three times.

### 2.4 Validation in soft cheese

Mexican soft cheese—queso fresco brand A—was obtained from a local grocery store and stored at 4 °C. The BAM culture method was used to confirm the absence of naturally occurring *L. monocytogenes* (Hitchins et al., 2022). The Aerobic Plate Count (APC) analysis was performed to assess the background flora level (Zhang et al., 2026). The queso fresco sample was bulk inoculated at low (i.e., fractional) and high (1 log higher than fractional) levels. Another portion (125 g) was used as the uninoculated control.

For low-level inoculation, 700 μL of diluted *L. monocytogenes* CFSAN011016 culture in BHI was added dropwise to 550 g of queso fresco, reaching 0.2–2 CFU/25 g test portion. The bulk sample was subjected to vigorous mixing, which included hand-massaging, splitting, and stomaching at 250 rpm for 30 s, followed by combining and hand-massaging, then splitting and stomaching again, and combining for the final hand-massaging. Additionally, three 125 g portions and three 5 g portions were set aside for the most-probable-number (MPN) analysis. For high-level inoculation, 200 μL of diluted *L. monocytogenes* CFSAN011016 culture in BHI was added to 150 g of queso fresco, reaching 2–20 CFU/25 g test portion. Both high-level inoculated and uninoculated controls were hand-massaged, stomached at 250 rpm for 30 s, and hand-massaged again. All bulk samples, including low-level and high-level inoculations, the uninoculated control, and MPN portions, were aged at 2 °C–8 °C for 48–72 h before testing.

On the day of testing, a three-level MPN analysis was performed on the reserved low-level MPN portions (Blodgett, 2023). All bulk queso fresco samples were portioned out to 25 g each, 30 in total, consisting of 5 uninoculated, 20 low-level inoculated, and 5 high-level inoculated test portions. Each test portion was analyzed using the BAM culture method (detailed below). DNA extraction using the DNeasy Blood & Tissue Kit (450 μL input volume; pretreatment for Gram-positive bacteria; 100 μL elution buffer) (Qiagen, Germantown, MD, United States) and the LAMP assay (described above) were performed on 48 h enrichments.

### 2.5 Validation in raw pet food

The test portions for raw pet food validation were prepared by a third-party laboratory (Q Laboratories, Cincinnati, OH, United States) per FDA specifications. Briefly, chicken-based frozen raw dog food was obtained from an online store and stored at −20 °C. The raw pet food sample was screened for the absence of naturally occurring *L. monocytogenes* using BAM culture (Hitchins et al., 2022), and APC was performed to assess the background flora level (Zhang et al., 2026). After storing at 4 °C overnight, the thawed raw pet food samples were portioned into bulk samples for low (1,000 g) and high (250 g) levels of inoculations, an uninoculated control (250 g), APC (200 g), and MPN portions (5 replicates of 50 g and 10 g for low-level inoculation and 5 replicates of 10 g and 5 g for high-level inoculation). The inoculum was prepared by propagating the *L. monocytogenes* ATCC 19111 strain from a stock culture stored at −70 °C onto a tryptic soy agar with 5% sheep blood (SBA) plate (Thermo Fisher Scientific) and incubated at 35 °C ± 1 °C for 24 ± 4 h. A single colony was transferred to BHI broth and incubated at 35 °C ± 1 °C for 24 ± 4 h. The cultures were then diluted in fresh BHI broth to the appropriate levels necessary for inoculation (0.2–2 CFU/test portion for the low level and 2–5 CFU/test portion for the high level). For inoculation, the diluted cultures were added dropwise to the bulk samples while mixing periodically in large sterile stainless-steel containers. Sterile spatulas were used to mix the bulk portions to ensure that the inoculum was evenly distributed. All bulk samples were apportioned into 25 g test portions, 30 in total, consisting of 5 uninoculated, 20 with low-level inoculation, and 5 with high-level inoculation. These test portions, along with the APC and MPN portions, were aged at −20 °C for at least 2 weeks before testing.

On the day of testing, a three-level MPN analysis was performed on the reserved MPN portions at both low and high levels (Blodgett, 2023), and APC was performed on the reserved APC portion (Zhang et al., 2026). Similar to the soft cheese validation, DNA extraction and LAMP assay were performed on 48 h enrichments. For DNA extraction, besides the Qiagen DNeasy Blood & Tissue Kit (450 μL input volume; 100 μL elution buffer), PrepMan Ultra Sample Preparation Reagent (1 mL input volume; 100 μL elution buffer; Thermo Fisher Scientific) was also used as described previously (Ge et al., 2019). When LAMP inhibition was observed, the PrepMan templates were diluted 1:10 for retesting.

### 2.6 The BAM reference culture method

**Figure 2:**
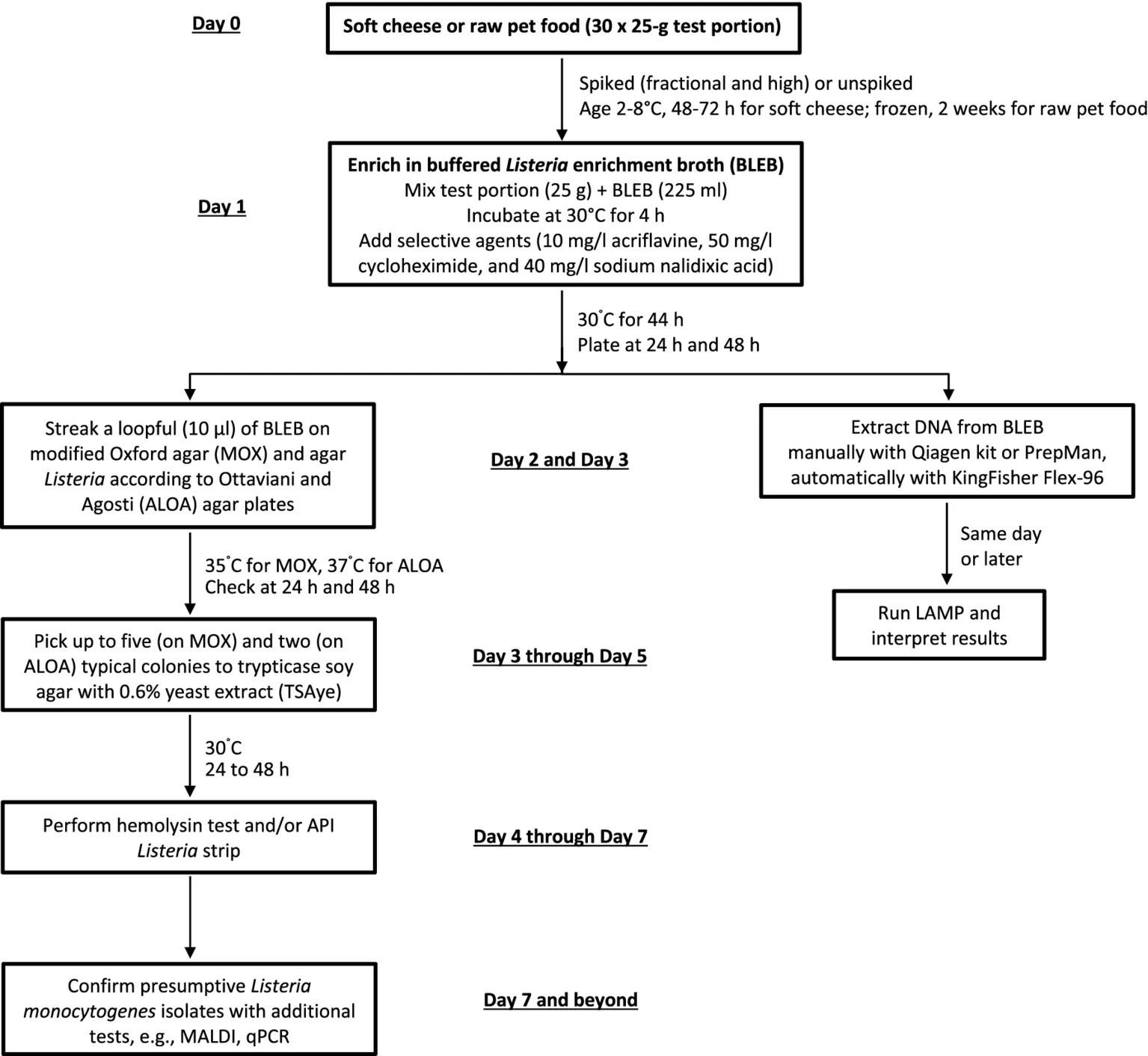
A schematic diagram showing the paired study design to validate the *Listeria monocytogenes* loop-mediated isothermal amplification (LAMP) assay in soft cheese and raw pet food using the U.S. Food and Drug Administration’s (FDA) *Bacteriological Analytical Manual* (BAM) Chapter 10 culture method as a reference.

The protocol detailed in the FDA’s BAM Chapter 10, Sections D through H (Hitchins et al., 2022), was followed. All media and reagents were obtained from Thermo Fisher Scientific, unless specified otherwise. As illustrated in Figure 2, 225 mL of buffered *Listeria* enrichment broth (BLEB) was added to each test portion, and after stomaching and thorough mixing, it was incubated at 30 °C for 4 h. Following the addition of selective agents for BLEB (10 mg/L acriflavin, 50 mg/L cycloheximide, and 40 mg/L sodium nalidixic acid; Hardy Diagnostics, Santa Maria, CA, United States), the incubation proceeded for an additional 44 h. The 24 h and 48 h enrichments were streaked onto modified Oxford agar (MOX; Hardy Diagnostics) and ALOA and incubated at 35 °C (for MOX) or 37 °C (for ALOA) for up to 48 h. Up to five (on MOX) or two (on ALOA) presumptive *L. monocytogenes* colonies were transferred to trypticase soy agar containing 0.6% yeast extract (TSAye) for growth. For confirmation, the hemolysin test and/or Analytical Profile Index (API) *Listeria* strips (BioMérieux) were performed, followed by the Bruker Biotyper method for *Listeria* spp., *L. monocytogenes*, and other Gram-positive bacteria (AOAC-OMA 2017.10).

### 2.7 Data analysis

Means and standard deviations of LAMP *T_p_* and *T_a_* were calculated using Microsoft Excel and compared among serotypes and lineages using analysis of variance followed by Tukey’s Honestly Significant Difference (HSD) post-hoc test using RStudio (version 4.5.2) with statistical significance at *p* < 0.05 (R Core Team, 2026). Differences between means were considered significant at *p* < 0.05. LODs were presented as the lowest numbers of *L. monocytogenes* cells that could be detected by the LAMP assay per reaction. In soft cheese and raw pet food validation trials, APC and MPN were calculated using the Aerobic Plate Count Density Estimator v0.6.0 (FDA, 2026b) and MPNcalc v1.7.0 (Ferguson and Ihrie, 2026), respectively. Comparisons between LAMP and BAM were expressed as the Relative Level of Detection (RLOD) using the RLOD program v4 (Wilrich, 2024). An acceptability limit (AL) of 1.5 was used, as both were paired studies (ISO, 2016).

## 3 Results

### 3.1 LAMP optimization

**Figure 3:**
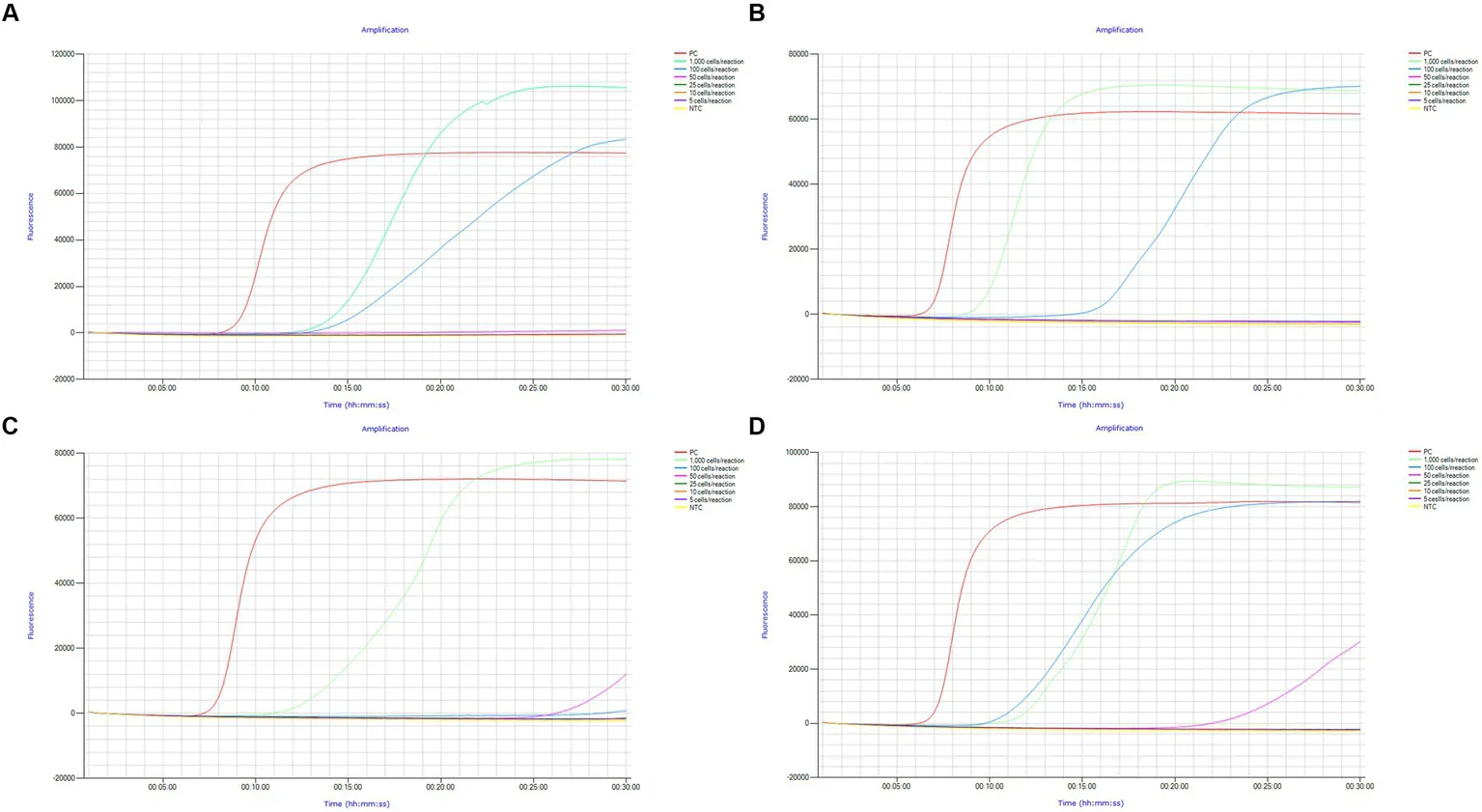
Optimization of the *Listeria monocytogenes* loop-mediated isothermal amplification (LAMP) assay using four primer mixes for testing a template series of *L. monocytogenes* ATCC 19115. **(A)** OptiGene Standard, **(B)** OptiGene High, **(C)** NEB WarmStart, and **(D)** Prototype. Samples are in this order: Positive control (PC), *L. monocytogenes* ATCC 19115 templates at concentrations 1,000, 100, 50, 25, 10, and 5 cells/reaction, and no-template control (NTC).

Figure 3 shows the representative LAMP amplification graphs generated using the four primer mixes when testing a *L. monocytogenes* ATCC 19115 template series (1,000, 100, 50, 25, 10, and 5 cells/reaction). All four primer mixes consistently (3 replicates) detected the 1,000 cells/reaction template with the fastest *T_p_* of 12.0 ± 0.7 min (prototype), while *T_p_* for the other primer mixes ranged from 15.4 ± 3.5 min (OptiGene High) to 16.6 ± 0.8 min (OptiGene Standard). Using the 30 min cutoff for *T_p_*, NEB WarmStart detected 100 cells/reaction template in 1 replicate (29.8 min), while OptiGene Standard and OptiGene High each detected it in 2 replicates, averaging 15.7 ± 0.9 min and 21.5 ± 5.7 min, respectively, and the prototype detected it in all 3 replicates (18.6 ± 9.4 min). For the 50 cells/reaction template, late amplifications (*T_p_* > 20 min) were noted, and the detection rate was 1 replicate each for OptiGene Standard (28.8 min), OptiGene High (29.8 min), and NEB WarmStart (29.8 min) and 2 replicates for the prototype (24.6 ± 4.6 min).

Running the Genie II instrument-programmed temperature gradient from 60 °C to 67 °C, corresponding to approximately 60 °C, 62 °C, 65 °C, and 67 °C in duplicate wells, the detection rates (using a more stringent *T_p_* cutoff of 20 min) for the 100 cells/reaction template in 10 reps were 70, 80, 80, and 40%, respectively (data not shown). For individual temperature runs from 60 °C to 65 °C using an abbreviated *L. monocytogenes* ATCC 19115 template series (100, 50, 25, 10, and 5 cells/reaction), detection down to the 5 cells/reaction template was observed at 60 °C and 62 °C, and detection rates for any amplification signals (mostly between 20 and 30 min) at that level (5 cells/reaction) increased as the temperature decreased from 63 °C (16.7%) to 60 °C (66.7%). Amplification signals for the 1 cell/reaction template were observed sporadically at both 61 °C and 62 °C with *T_p_* > 20 min. Nonetheless, using an abbreviated exclusivity panel at 61 °C and 62 °C, sporadic amplification signals were observed (data not shown). In contrast, the 65 °C run profile resulted in clean graphs (no amplification signals during the 30 min amplification stage) for all 51 strains in the exclusivity panel (detailed below). Thus, the LAMP assay using the prototypic primer mix and a reaction temperature of 65 °C was chosen.

### 3.2 Assay specificity

As shown in Table 1, LAMP detected 43 (out of 50, probability of detection [POD] of 86%) *L. monocytogenes* initial inclusivity templates (~10^0^–10^2^ cells/reaction). Testing the seven templates (C1-122, J1-049, J1-094, J1-158, J2-035, W1-111, and W1-112) belonging to various serotypes and lineages at concentrations higher than those used for initial inclusivity resulted in LAMP detection of all seven templates with positive detection starting at ~10^1^ cells/reaction (3 strains; C1-122, J1-049, and J1-094), ~10^2^ cells/reaction (2 strains; J1-158 and W1-112), and ~10^3^ cells/reaction (2 strains; J2-035 and W1-111), and consistent detection at concentrations above LOD (data not shown). Therefore, we added the corresponding *T_p_* and *T_a_* at 10^2^ cells/reaction to Table 1 (except for J2-035 and W1-111, where values for 10^3^ cells/reaction were used), suggesting 100% inclusivity. The *T_p_* for the 50 *L. monocytogenes* inclusivity panel ranged from 6.1 to 25.4 min (11.3 ± 3.9 min), and *T_a_* ranged from 82.0 °C to 84.9 °C (82.6 °C ± 0.4 °C).

Among 38 *L. monocytogenes* strains belonging to the three major serotypes tested (1/2a, 1/2b, and 4b), *T_p_* was the lowest for 1/2a (8.5 ± 1.4 min; *n* = 11), significantly lower than that for 4b (12.1 ± 3.2 min; *n* = 22) (*p* = 0.0017) but not for 1/2b (11.2 ± 1.4 min; *n* = 5) (*p* = 0.1497) (data not shown). All strains belonging to serotype 1/2a were detected using the initial inclusivity templates, whereas 1/2b strains were not detected in one template and 4b strains were not detected in two templates. The other four strains that could not be detected using the initial inclusivity templates belonged to serotypes 1/2c, 3c, 4a, and 4c. There was also a significant difference (*p* = 0.0001) in *T_p_* between strains in lineages I (11.9 ± 2.7 min; *n* = 28) and II (8.5 ± 1.5 min; *n* = 14); understandably, most 1/2a strains belonged to lineage II, whereas 1/2b and 4b strains belonged to lineage I. Interestingly, there were also significant differences (*p* < 0.05) in *T*_*a*_ among serotypes (1/2a vs. 1/2b, and 1/2a vs. 4b) and lineages (I vs. II) (data not shown).

For the 51 exclusivity strains, consisting of 20 non-*L. monocytogenes Listeria* and 31 non-*Listeria* strains at ~10^6^ cells/reaction concentration (growth limits of the strains per the FDA guidelines (FDA, 2019), negative results were obtained for all of them using LAMP, suggesting 100% exclusivity.

### 3.3 Assay sensitivity

**Figure 4:**
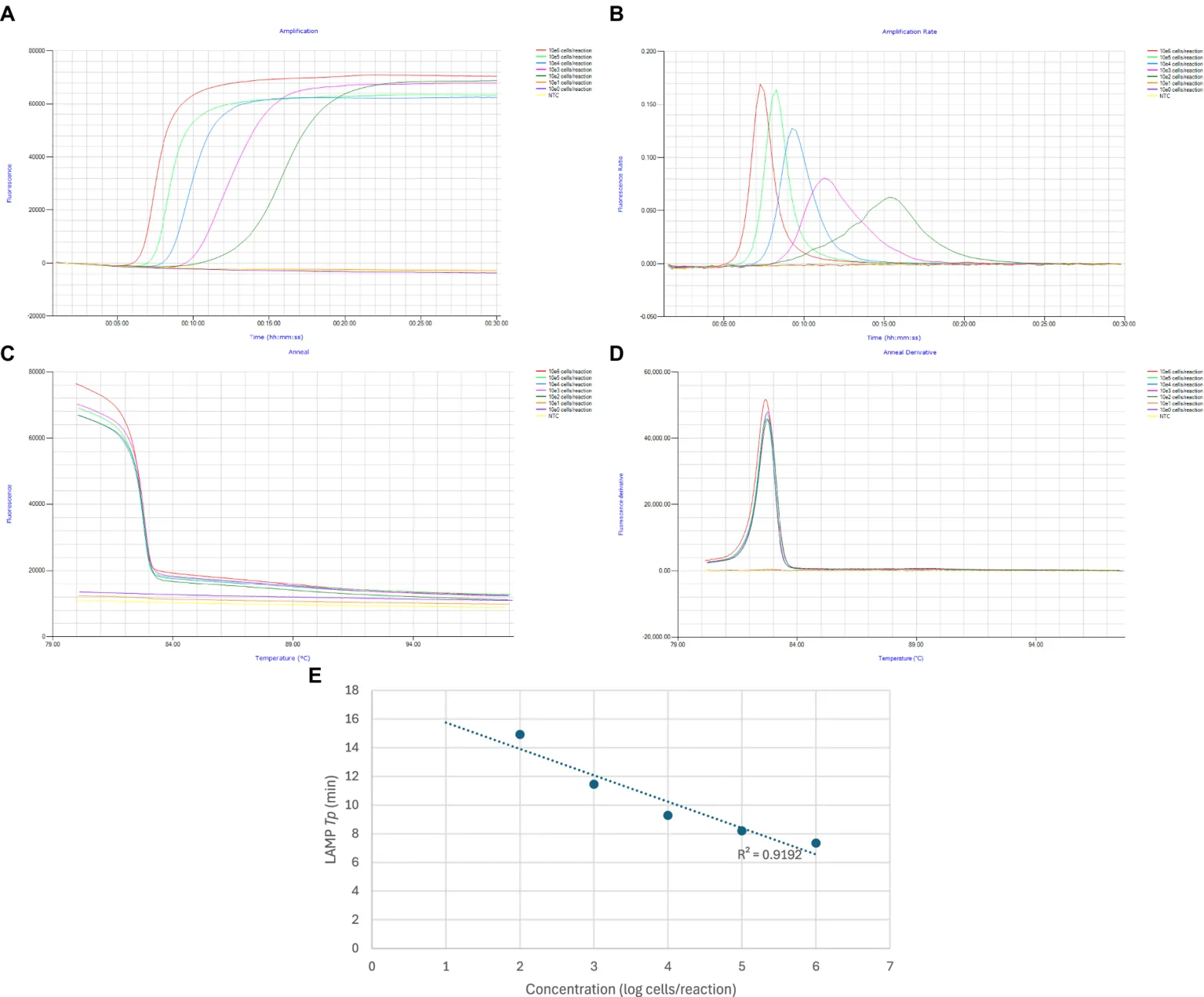
Representative *Listeria monocytogenes* loop-mediated isothermal amplification (LAMP) sensitivity graphs when testing a template series of *L. monocytogenes* ATCC 19115. **(A)** LAMP amplification graph; **(B)** LAMP amplification rate curve; **(C)** LAMP anneal graph; **(D)** LAMP anneal derivative curve; and **(E)** a standard curve generated based on triplicate LAMP sensitivity testing. Samples 1 to 7 correspond to 10-fold serial dilutions of *L. monocytogenes* ATCC 19115 templates ranging from 1 × 10^6^ to 1 cell/reaction; and sample 8 is no-template control (NTC).

Figure 4 shows a representative LAMP amplification graph, amplification rate curve, anneal graph, and anneal derivative curve when testing 10-fold serial dilutions of *L. monocytogenes* ATCC 19115 ranging from 1 × 10^6^ to 1 cell/reaction (Figures 4A–D). In all three replicates, the LAMP assay consistently detected down to the 100 cells/reaction level with *T_p_* ranging from 7.3 ± 0.03 min at 1 × 10^6^ cells/reaction to 14.9 ± 1.9 min at 100 cells/reaction and an overall *T_a_* averaging 82.7 °C ± 0.2 °C. A standard curve generated based on these triplicate runs showed a good linear relationship, with a correlation coefficient (*R*^2^) of 0.919 (Figure 4E).

As indicated earlier, seven templates (C1-122, J1-049, J1-094, J1-158, J2-035, W1-111, and W1-112), that failed initial inclusivity and belonged to various serotypes and lineages, were tested by serial dilutions. The LODs for these strains ranged from approximately 10^1^ cells/reaction (3 strains; C1-122, J1-049, and J1-094) to ~10^2^ cells/reaction (2 strains; J1-158 and W1-112) and to ~10^3^ cells/reaction (2 strains; J2-035 and W1-111).

### 3.4 Validation in soft cheese

APC for the queso fresco sample was 13 CFU/g. When inoculated with *L. monocytogenes* CFSAN011016 at low (1.1 CFU/25 g; 0.7 MPN/25 g after aging) and high (10.8 CFU/25 g) concentrations, the validation study yielded satisfactory results (Table 2). None of the uninoculated control portions yielded positive results by LAMP or BAM. All 48 h enrichments of test portions with high-level inoculation tested positive by the LAMP assay, all of which were confirmed by BAM. For test portions inoculated at the low concentration, the POD was 45% for both LAMP (average *T_p_* of 8.1 ± 0.4 min) and BAM, well within the fractional detection range targeted (25–75%). Notably, consistent test-portion-level agreements (100%) were observed between LAMP and BAM at high-level inoculation, low-level inoculation, and uninoculated controls.

**Table 2:** Comparison of the *Listeria monocytogenes* loop-mediated isothermal amplification (LAMP) assay (analysis of 48 h enrichments) and the U.S. Food and Drug Administration’s (FDA) *Bacteriological Analytical Manual* (BAM) Chapter 10 culture method for detecting low-level initial contaminations of *L. monocytogenes* strains in 25 g test portions of soft cheese and raw pet food.

Food matrix	APC (CFU/g)	*L. monocytogenes* strain	Contamination level	Concentration (per 25 g)	No. of test portions	No. (%) of positive test portions		RLOD (confidence interval)
						BAM	LAMP	
Queso fresco-type soft cheese	13	CFSAN011016	Uninoculated	NA	5	0 (0)	0 (0)	1 (0.436–2.296)
			Low	1.1 CFU (0.7 MPN)	20	9 (45)	9 (45)	
			High	10.8 CFU	5	5 (100)	5 (100)	
Chicken-based raw dog food	3.4 × 10^5^	ATCC 19111	Uninoculated	NA	5	0 (0)	0 (0)	1.349 (0.517–3.517)
			Low	1 CFU (0.75 MPN)	20	9 (45)	7 (35)	
			High	5.4 CFU (2.75 MPN)	5	5 (100)	5 (100)	

The Qiagen kit was used for DNA extraction in the soft cheese trial, whereas both Qiagen and PrepMan kits were used in the raw pet food trial. There were complete test-portion-level agreements (100%) in the soft cheese trial. In the raw pet food trial, two test portions were positive by either the Qiagen kit extraction or the PrepMan kit extraction, but not both. Both LAMP and RLOD results were obtained from a single kit, either the Qiagen kit or the PrepMan kit.

The RLOD between the LAMP assay (analysis of 48 h enrichment) and BAM was 1, suggesting that the two methods were equally sensitive at detecting low-level contamination of *L. monocytogenes* in soft cheese. This RLOD value fell within the acceptability limit of 1.5 for a paired study (ISO, 2016).

### 3.5 Validation in raw pet food

APC for the raw pet food sample was 3.4 × 10^5^ CFU/g. As shown in Table 2, none of the uninoculated control test portions yielded positive results by LAMP or BAM. All 48 h test portions inoculated with *L. monocytogenes* ATCC 19111 at the high concentration (5.4 CFU/25 g; 2.75 MPN/25 g after aging) tested positive by LAMP, all of which were confirmed by BAM. For test portions inoculated at the low concentration (1 CFU/25 g; 0.75 MPN/25 g after aging), the POD was 45% for BAM and 35% for LAMP with either Qiagen (average *T_p_* of 19.0 ± 4.6 min) or PrepMan (average *T_p_* of 15.4 ± 4.0 min) kits, both of which were within the fractional detection range targeted (25–75%). For low-level inoculation, two test portions were positive by either Qiagen kit extraction or PrepMan kit extraction, but not both. It is important to note that initial testing with PrepMan-extracted DNA had strong inhibitions with unusual amplification graphs but normal anneal graphs. The final data were obtained using 1:10 diluted PrepMan-prepared templates.

The RLOD between the LAMP assay (analysis of 48 h enrichments with DNA extracted manually with the Qiagen kit or the PrepMan kit) and BAM was 1.349, within the acceptability limit of 1.5 for a paired study (ISO, 2016). This suggested that LAMP (analysis of 48 h enrichments using either DNA extraction method) was as sensitive as BAM in detecting low-level contamination of *L. monocytogenes* in raw pet food.

## 4 Discussion

We developed and optimized a *hlyA*-based *L. monocytogenes* LAMP assay and performed SLV in both human and animal food matrices, represented by queso fresco-type soft cheese and chicken-based raw dog food. Following the FDA’s Microbiological Method Validation Guidelines (FDA, 2019), both SLV studies showed that the LAMP assay was rapid, specific, and sensitive and performed comparably to the FDA’s BAM reference method (Hitchins et al., 2022).

As a promising alternative to real-time PCR, LAMP is amenable to using the same target genes for assay design as PCR-based assays (Chen et al., 2017; Kumar et al., 2015), such as *hlyA* (listeriolysin O) (Zbrun et al., 2024; Rodriguez-Lazaro et al., 2004; Klein and Juneja, 1997), *iap* (invasion-associated protein P60) (Klein and Juneja, 1997; Bubert et al., 1992; Chen Y. et al., 2011; Eiken Chemical Co. Ltd., 2013), *prfA* (positive regulator factor A) (Klein and Juneja, 1997; Jung et al., 2009), *inlA*/*inlB* (internalins A and B) (Jung et al., 2003), *mpl* (metalloproteinase) (Tao et al., 2015), *lmo0733* (aminopeptidase) (Liu et al., 2004), *lmo2234* (putative protein) (Zhang et al., 2004), *ssrA* (small, stable RNA) (O’Grady et al., 2008), and the 16S rRNA gene (Somer and Kashi, 2003; Wiedmann et al., 1993). Multiple LAMP assays targeting these genes have been reported (Rivas-Macho et al., 2023; Ledlod et al., 2020; Wang et al., 2011; Garrido-Maestu et al., 2018; Wang et al., 2010; Wang et al., 2015; Busch et al., 2022; Cho et al., 2014). We chose the most widely used *hlyA* gene as the basis for our LAMP assay design because it is a key *L. monocytogenes* virulence factor involved in the lysis of the host vacuolar membrane and is present in all *L. monocytogenes* strains (Cossart et al., 1989). As a companion to this study, Mallmann et al. (2026) evaluated 14 *hlyA*-based LAMP assays and showed that these assays had the overall best performance.

We chose to use the OptiGene ISO-004 kit in developing our LAMP assay, as it was recommended by the Genie II manufacturer (OptiGene) and was found to be the best performer independently evaluated by a recent study (Costa-Ribeiro et al., 2024). To optimize the LAMP assay, we first evaluated it using a primer mix (prototype) previously optimized for *Salmonella* detection (Chen S. et al., 2011). Compared to three other primer mixes, two recommended by the Genie II manufacturer (OptiGene Standard and OptiGene High) and another widely used LAMP kit (NEB WarmStart), we showed that the prototypic primer mix (0.1 μM F3/B3, 1.8 μM FIP/BIP, and 1 μM forward loop primer [LF; not used in this assay]/LB), optimized previously in the *Salmonella* LAMP assay (Chen S. et al., 2011), had a superior performance over the other three mixes (Figure 3), highlighting its broad applicability. Researchers developing LAMP assays for other microorganisms or associated genetic markers may consider adopting this optimized primer mix in their studies. We then attempted to optimize the LAMP reaction temperatures outside of the standard 65 °C, the optimum temperature for the strand-displacing GspSSD2.0 LF DNA polymerase used in this kit. The rationale was considering the low G/C content (~38%) of the *L. monocytogenes* genome (Glaser et al., 2001). Our results showed that at LAMP reaction temperatures lower than 63 °C, exclusivity of the assay was sacrificed, despite an increase in sensitivity (down to 1 cell/reaction), thus not warranting the switch. As a reference, earlier studies optimizing amplification temperatures for *hlyA*-based *L. monocytogenes* LAMP found 63 °C–65 °C to be optimal (Rivas-Macho et al., 2023; Garrido-Maestu et al., 2018; Tang et al., 2011; Wang et al., 2012; Huang et al., 2015; Ma et al., 2019; Wan et al., 2012).

Our newly designed *L. monocytogenes* LAMP assay was 100% specific among 101 strains (inclusivity panel, 50 *L. monocytogenes* of 10 serotypes; exclusivity panel, 20 non-*L. monocytogenes* of 5 *Listeria* spp. and 31 non-*Listeria* of 13 genera). This high level of specificity corroborated previously reported *hlyA*-based *L. monocytogenes* LAMP assays (Rivas-Macho et al., 2023; Wang et al., 2011; Garrido-Maestu et al., 2018; Tang et al., 2011; Wang et al., 2012; Huang et al., 2015; Ma et al., 2019; Wan et al., 2012; Pisamayarom et al., 2017; Shan et al., 2012; Ye et al., 2015; Cheng et al., 2016; Wachiralurpan et al., 2017; Tirloni et al., 2017), although limited numbers of strains were used in most studies. Exceptions are Wang et al. (2012), who tested 182 *L. monocytogenes* food isolates with 96.7% inclusivity (6 strains were not detected), whereas Shan et al. (2012), Ye et al. (2015), and Wan et al. (2012) tested 92, 58, and 45 *L. monocytogenes* strains, respectively, all with 100% inclusivity. Concentrations of specific DNA templates used in these studies were unspecified except for one study by Cheng et al. (2016), which used 100 ng/μL (~10^8^ cells/reaction) for both inclusivity and exclusivity. In the present study, the inclusivity templates were initially prepared at *ca.* 10^3^–10^5^ CFU/mL (i.e., ~10^0^–10^2^ cells/reaction), in reference to a previously validated *Salmonella* LAMP assay (Domesle et al., 2018). Due to the higher LOD of this *L. monocytogenes* LAMP assay (100 cells/reaction using ATCC 19115) than that of the *Salmonella* assay (1 cell/reaction), further testing of seven inclusivity templates that failed initial testing at higher concentrations (i.e., 10–100-fold of the LOD_50_ per the FDA guidelines (FDA, 2019)) confirmed the positive detection of all seven strains. This corroborated a recent report showing the need to adjust the inclusivity template concentration for one strain due to a lower sensitivity (higher LOD) (Ge et al., 2025). Nonetheless, our testing showed that the LOD for the two strains was higher (~10^3^ cells/reaction), which may be attributed to sequence variation in the primer-binding regions of the *hlyA* target gene. Furthermore, we observed significant differences in *T_p_* among *L. monocytogenes* serotypes/lineages, with those belonging to serotype 1/2a and lineage II having the fastest detection (*p* < 0.05). This may be caused by *hlyA* gene sequence variations across *L. monocytogenes* serotypes/lineages (Rasmussen et al., 1995), whereas the reference genome used for primer design, the *L. monocytogenes* EGD-e chromosome, belongs to serotype 1/2a and lineage II (Glaser et al., 2001).

The LOD for our newly developed *L. monocytogenes* LAMP assay was 100 cells/reaction in pure culture, based on triplicate sensitivity testing of *L. monocytogenes* ATCC 19115. This level of sensitivity fell within the range (1 cell/reaction to 3.7 × 10^3^ CFU/mL) reported previously among *hlyA*-based *L. monocytogenes* LAMP assays (Rivas-Macho et al., 2023; Wang et al., 2011; Garrido-Maestu et al., 2018; Tang et al., 2011; Wang et al., 2012; Huang et al., 2015; Ma et al., 2019; Wan et al., 2012; Pisamayarom et al., 2017; Shan et al., 2012; Ye et al., 2015; Cheng et al., 2016; Wachiralurpan et al., 2017; Tirloni et al., 2017). The *L. monocytogenes* LAMP assay developed in this study demonstrated good linearity for *L. monocytogenes* ATCC 19115 DNA templates ranging from 10^6^ to 10^2^ cells/reaction (Figure 4E), suggesting a strong quantification capability, as similarly reported in earlier studies (Rivas-Macho et al., 2023; Shan et al., 2012; Cheng et al., 2016).

The application of *hlyA*-based LAMP assays for *L. monocytogenes* detection in food, first reported as a proof-of-concept in chicken in 2011 (Wang et al., 2011; Tang et al., 2011), has since expanded to multiple platforms and matrices (Rivas-Macho et al., 2023; Tirloni et al., 2017; Wu et al., 2014; Birmpa et al., 2015; Xiong et al., 2016; Fiore et al., 2023; Wachiralurpan et al., 2018). The platforms used to monitor LAMP reactions included naked-eye observation, gel electrophoresis, turbidity, fluorescence, bioluminescence, electrochemical methods, and microfluidics. For contamination-proof, closed-tube monitoring of LAMP reactions, real-time fluorescence has gained popularity, especially with the advent of small-footprint, portable, and low-maintenance instruments such as Genie II. Fluorescence signals are generated in real-time, as displayed in amplification graphs and amplification rate curves, followed by a specificity check on anneal graphs and anneal derivative curves (Figure 3). Such user-friendly characteristics will greatly facilitate the future adoption of this newly developed *L. monocytogenes* LAMP assay. In food matrices, 48 h enrichments are commonly applied, making the detection of quite low-level initial *L. monocytogenes* contaminations (~1 cell/25 g test portion) feasible, as demonstrated in our testing in soft cheese and raw pet food and reported previously (Rivas-Macho et al., 2023).

Despite major efforts in developing and evaluating *L. monocytogenes* LAMP assays in food matrices, few studies have validated these assays in human food (none in animal food or compatible with the BAM workflow, to our knowledge) following the well-established AOAC, FDA, and/or ISO guidelines (FDA, 2019; AOAC, 2023; ISO, 2016). Following AOAC Performance Tested Methods (PTM) certification in eight human food matrices (beef hot dogs, deli turkey, cold-smoked salmon, full-fat cottage cheese, chocolate milk, bagged raw spinach, romaine lettuce, and cantaloupe) and on two environmental surfaces (sealed concrete and stainless steel), a multi-laboratory collaborative study in 2015 (Bird et al., 2015) validated the 3 M Molecular Detection Assay (MDA) *Listeria monocytogenes* (Neogen, Lansing, MI, United States), a bioluminescence-based LAMP assay, in deli turkey and full-fat cottage cheese and showed similar performance compared to culture-based methods—USDA’s MLG 8.09 (2011) method or AOAC-OMA 993.12. Another multi-laboratory collaborative study in 2017 (Bird et al., 2017) validated the modified version of the MDA assay, MDA2 *Listeria mono*, in deli turkey and raw chicken breast filet, which showed similar or higher detection rates in the LAMP-based MDA2 compared to the USDA’s MLG 8.09 method. In the present study, the SLV validation of the newly developed *L. monocytogenes* LAMP assay in one human food (queso fresco-type soft cheese) and one animal food (chicken-based raw dog food) yielded satisfactory results, although we acknowledge that a single strain was used in both validation trials and only one type of soft cheese or raw pet food was used, not accounting for variability across strains or product types. Compared to the BAM culture method, the RLOD for LAMP with the Qiagen kit-extracted 48 h soft cheese enrichments was 1 (same POD), and with either the Qiagen kit or the PrepMan kit-extracted 48 h raw pet food enrichments, it was 1.349; both fell within the acceptability limit (1.5) for a paired study. This suggested that LAMP (analysis of 48 h enrichments) was as sensitive as the FDA’s BAM culture method in detecting low-level initial *L. monocytogenes* contamination (~1 cell/25 g test portion) in both soft cheese and raw pet food, with reliable results available at least 3 days sooner.

Consistent test-portion-level agreements (100%) were observed between LAMP and BAM in the soft cheese validation trial, which also had faster *T_p_* values compared to those in the raw pet food trial (8.1 ± 0.4 min vs. 19.0 ± 4.6 min by the Qiagen kit and 15.4 ± 4.0 min by the PrepMan kit). This may be attributed to the lower background flora in the former (13 CFU/g vs. 3.4 × 10^5^ CFU/g). Notably, PrepMan extraction resulted in faster *T_p_* values than those extracted by the Qiagen kit in the raw pet food trial, despite the inhibition observed when tested directly by LAMP. The PrepMan kit is a rapid and simple DNA extraction method compared to the Qiagen kit, and its promising results warrant further exploration.

The importance of *L. monocytogenes* in food safety, particularly its ability to persist in food processing environments for years, combined with its psychrotrophic growth characteristics, necessitates comprehensive environmental monitoring programs and product testing and stringent control measures throughout the food supply chain. The LAMP assay validated in this study, in both soft cheese and raw pet food, and compatible with the BAM workflow and multiple DNA extraction methods, presents a valuable tool as a rapid, reliable, and robust method for routine screening of *L. monocytogenes* in these commodities when used in conjunction with BAM culture.

## 5 Conclusion

This method development and SLV validation study indicated that our newly developed *L. monocytogenes* LAMP assay (analyzing 48 h enrichments with multiple DNA extraction methods) was rapid (within 30 min of assay time), specific, and sensitive for detecting low-level *L. monocytogenes* contamination (~1 CFU/25 g test portion) in both soft cheese and raw pet food. With further SLV studies or matrix extension in additional human and animal food matrices and multi-laboratory validation, it may be adopted in the FDA’s field laboratories for routine screening of *L. monocytogenes* in these commodities and used in conjunction with BAM culture to ensure product safety and protect public health.

## Data Availability

The datasets presented in this study are included in the article and further inquiries can be directed to the corresponding author.
